# Plasma copeptin and markers of arterial disorder in patients with type 2 diabetes, a cross-sectional study

**DOI:** 10.1186/s12933-024-02291-2

**Published:** 2024-06-12

**Authors:** Lee Ti Davidson, Jan Engvall, Simona I. Chisalita, Carl Johan Östgren, Fredrik H. Nyström

**Affiliations:** 1 Department of Emergency Medicine in Linköping, Department of Biomedical and Clinical Sciences, Linköping University, 581 83 Linköping, Sweden; 2 Department of Clinical Physiology in Linköping, Department of Health, Medicine and Caring Sciences, Linköping University, Linköping, Sweden; 3 Department of Endocrinology in Linköping, Department of Health, Medicine and Caring Sciences, Linköping University, Linköping, Sweden; 4 Division of Prevention, Rehabilitation and Community Medicine, Department of Health, Medicine and Caring Sciences, Linköping University, Linköping, Sweden; 5 Division of Diagnostics and Specialist Medicine, Department of Health, Medicine and Caring Sciences, Linköping University, Linköping, Sweden; 6 Centre for Medical Image Science and Visualization (CMIV), Linköping University, Linköping, Sweden

**Keywords:** Type 2 diabetes, Copeptin, MRproADM, MRproANP, Toe brachial index, Pulse wave velocity, Cardiovascular disease

## Abstract

**Objectives:**

There is currently limited understanding of the relationship between copeptin, the midregional portion of proadrenomedullin (MRproADM) and the midregional fragment of the N-terminal of proatrial natriuretic peptide (MRproANP), and arterial disorders. Toe brachial index (TBI) and aortic pulse wave velocity (aPWV) are established parameters for detecting arterial disorders. This study evaluated whether copeptin, MRproADM, and MRproANP were associated with TBI and aPWV in patients with type 2 diabetes with no history of cardiovascular disease (CVD).

**Methods:**

In the CARDIPP study, a cross-sectional analysis of 519 patients with type 2 diabetes aged 55–65 years with no history of CVD at baseline, had complete data on copeptin, MRproADM, MRproANP, TBI, and aPWV was performed. Linear regression analysis was used to investigate the associations between conventional CVD risk factors, copeptin, MRproADM, MRproANP, TBI, and aPWV.

**Results:**

Copeptin was associated with TBI (β–0.0020, CI–0.0035– (–0.0005), *p* = 0.010) and aPWV (β 0.023, CI 0.002–0.044, *p* = 0.035). These associations were independent of age, sex, diabetes duration, mean 24-hour ambulatory systolic blood pressure, glycated hemoglobin A1c, total cholesterol, estimated glomerular filtration rate, body mass index, and active smoking.

**Conclusions:**

Plasma copeptin may be a helpful surrogate for identifying individuals at higher risk for arterial disorders.

**Trial Registration:**

ClinicalTrials.gov identifier NCT010497377.

**Supplementary Information:**

The online version contains supplementary material available at 10.1186/s12933-024-02291-2.

## Introduction

Patients with type 2 diabetes mellitus (DM) are at risk of developing cardiovascular disease (CVD), a leading cause of morbidity and mortality. Therefore, it is crucial to identify new and clinically useful cardiovascular (CV) risk markers to prevent overt CVD. In addition to established CV risk factors such as age, sex, smoking status, hypertension, and hypercholesterolemia, type 2 DM is also a significant risk factor for arterial stiffness [1]. Arterial stiffness occurs due to structural changes in the wall and vascular smooth muscle tone [2–4]. The currently preferred method for assessing arterial stiffness is to measure the aortic pulse wave velocity (aPWV), which indicates arterial stiffness in the proximal arteries. However, the aPWV does not evaluate the distal arteries [5]. On the other hand, due to the calcification of the media of arteries, the toe-brachial index (TBI) has been proposed as a better index for evaluating the peripheral circulation in patients with DM than the ankle-brachial index (ABI) [6, 7]. However, such vascular examinations are generally only available to primary care physicians after referral to a vascular laboratory.

Midregional proadrenomedullin (MRproADM) is the stable part of the pro-hormone adrenomedullin (ADM), a vasoactive peptide expressed mainly by vascular endothelial cells [8]. It regulates fluid-electrolyte homeostasis by acting on the renin-angiotensin-aldosterone system (RAAS) and hypothalamic-pituitary-adrenal axis [9]. Midregional proatrial natriuretic peptide (MRproANP) is a pro-hormone of the atrial natriuretic peptide (ANP) produced in the cardiac atrium that can induce vasodilatation, natriuresis and diuresis while inhibiting both the RAAS and the sympathetic nervous system [10, 11]. High concentrations of plasma arginine vasopressin (AVP) can stimulate V1a receptors [12], which can contribute to CV complications [13]. Copeptin, a stable peptide of AVP, is secreted in equimolar amounts to AVP from the pituitary gland in response to various stress-related stimuli [14].

Elevated levels of MRproADM, MRproANP, and copeptin are linked to vascular endothelial dysfunction [15–19] and have been found to correlate with arterial disease. However, studies on this topic are still scarce. This study aimed to investigate the association of these biomarkers with markers of arterial disorder, as assessed by vascular examination by aPWV and TBI, in patients with type 2 DM with no history of CVD or known peripheral arterial disease (PAD).

## Patients and methods

### Study population

A prospective observational cohort study called Cardiovascular Risk Factors in Patients with Diabetes - a Prospective Study in Primary Care (CARDIPP) was conducted to explore the impact of CV risk factors in patients with type 2 DM. A total of 761 patients aged 55–65 years with type 2 DM were consecutively recruited by specially trained diabetes care nurses during their usual annual follow-ups at 22 primary healthcare centers in the counties of Östergötland and Jönköping, Sweden. The study was carried out between 2005 and 2008. Patients with type 2 DM were identified on recorded medical diagnosis based on criteria recommended by the World Health Organization (WHO) in 1998. This study did not include patient with type 1 diabetes or gestational diabetes. The only exclusion criterion was having a severe physical or mental disease with a short life expectancy. For this analysis, we excluded 242 patients from the total CARDIPP cohort of 761. This exclusion was due to missing baseline data for aPWV (*n* = 59), TBI (*n* = 20), copeptin (*n* = 80), MRproADM (*n* = 79), or MRproANP (*n* = 71) measurements; known ischemic heart disease or stroke based on medical records and self-completed questionnaires (*n* = 118); or missing baseline data concerning previous CVD status (*n* = 4). After applying these criteria, we obtained a study sample of 519 participants with type 2 DM, accounting for any overlap between these exclusion criteria.

### Laboratory analyses

Blood samples were collected in the morning after a 10-hour overnight fast. Local laboratories analyzed routine tests, including hemoglobin A1c (HbA1c) and serum lipids. The local laboratories were accredited according to the international standard ISO-IEC 15,189. HbA1c was analyzed according to the Swedish Mono-S HPLC standard and converted into International Federation of Clinical Chemistry and Laboratory Medicine (IFCC) units (mmol/mol). The glomerular filtration rate (eGFR) was estimated using the Modification of Diet in Renal Disease (MDRD) formula by combining the creatinine values with age, sex, and ethnicity. Blood samples were frozen for later analysis. A highly sensitive time-resolved amplified cryptate emission technology assay (B.R.A.H.M.S, KRYPTOR, AG, Hennigsdorf, Germany) was used to analyze plasma MRproADM, MRproANP, and copeptin. The analytical detection limits for MRproADM, MRproANP, and copeptin were 0.04 nmol/L, 30.8 pmol/L, and 1.7 pmol/L, respectively. The inter-assay variability was 3.3%, 3.0%, and 5.2% for MRproADM, MRproANP, and copeptin, respectively.

### Anthropometric measurements

Body mass index (BMI) was calculated from the measurements of weight and height by nurses, primarily dedicated to treating diabetes at primary care centers, with the patients wearing light indoor clothing.

### 24-hour ambulatory systolic blood pressure

Ambulatory blood pressure measurement devices (Spacelab 90,217, Spacelabs Inc., Redmond, Washington, USA) were set to measure the blood pressure (BP) at 20-minute intervals for 24 h, as described in detail earlier [20].

### Toe brachial index

The method used to measure TBI was previously described [21]. In brief, toe pressure was measured on both sides using a strain-gauge technique (Medimatic, Hellerup, Denmark) after 10 min of rest. Brachial systolic blood pressure (SBP) was measured on both arms using an automated oscillometric device (Dinamap PRO 200 Monitor, Critikon, Tampa, FL, US). The TBI ratio was obtained by dividing the systolic toe pressure by the brachial SBP. This study used the lowest TBI ratio from either the left or right side for further analysis.

### Aortic pulse wave velocity

The aPWV was measured using an electrocardiogram-gated pulse wave analysis of the carotid and femoral arteries. This was performed with a Millar pressure tonometer and the SphygmoCor system (Model MM3, AtCor Medical, Sydney, Australia), as previously described [22]. The pulse wave transit time was calculated by subtracting the time between the ECG R-wave and the arrival of the pulse wave to the carotid measurement site from the time between the ECG R-wave and the appearance of the pulse wave to the femoral measurement site. The surface distance was defined as the distance between the suprasternal notch and the femoral measurement site (multiplied by 0.8), subtracted by the distance between the suprasternal notch and the carotid measurement site (multiplied by 0.8). The surface distance was divided by the pulse wave transit time to calculate the aPWV. All applanation tonometric investigations were performed at the University Hospital in Linköping or the County Hospital Ryhov, Jönköping, Sweden. Applanation tonometry measurements were made in duplicate and reported as the means of the two measurements.

### Ethics

All individuals provided written informed consent before participating. The Regional Ethical Review Board approved the study, DNR M26-05. The study protocol adhered to the principles expressed in the Declaration of Helsinki.

### Statistics

The statistical analyses were performed using SPSS software (IBM SPSS Statistics 29, Chicago, IL, USA). A two-sided *p* value less than 0.05 was considered to indicate statistical significance in all analyses. Unless otherwise stated, the values are presented as the means ± standard deviations or number of cases (percentage). The t test or Mann − Whitney U test was used for continuous variables to analyze the between-group differences in baseline characteristics by sex, whereas the chi − squared test was used for categorical data. Conventional risk factors for CVD were used as covariates in the adjusted multivariable model. These included sex, age, diabetes duration, BMI, smoking status, HbA1c-IFFC, total cholesterol, eGFR, and mean 24-hour ambulatory SBP. We assessed the associations of the conventional risk factors for CVD and the biomarkers MRproADM, MRproANP, and copeptin with aPWV and TBI by performing linear regression analysis. We started with univariable analysis followed by multivariable analysis, which included all CVD risk factors and each biomarker. Finally, we conducted a multivariable linear regression analysis that included all conventional CVD risk factors and all biomarkers, with aPWV and TBI as dependent variables. Correlation analysis using variance inflation factors explored confounding from traditional CVD risk factors and stress biomarkers. No collinearities of importance were noted in any of the models. For the multivariable regression analysis with a sample size of 519 and an estimated Cohen’s effect of at least 0.35, the power calculated to show a significant association with a *p* value ≤ 0.05 between the studied blood markers, adjusted for CVD risk factors, and TBI and aPWV was > 0.99.

### Result

Table 1 presents the baseline characteristics of 519 patients with no known CVD history and had complete data on TBI, aPWV, copeptin, MRproADM, and MRproANP. The study population primarily comprised male participants (63%) with a lower BMI, a lower frequency of diuretic use, lower cholesterol, and lower MRproADM levels than did the female participants. The male participants had higher eGFRs and copeptin levels than the female participants.

### Toe brachial index

This study found significant associations between age, diabetes duration, BMI, current smoking status, eGFR, mean 24-hour SBP, HbA1c, and copeptin to TBI (Table 2). However, after adjustment, only diabetes duration and copeptin remained significantly associated with TBI (Tables 3 and 4). There were no sex differences, or associations with TBI that could be established by univariable or multivariable analysis (as presented in Tables 1 and 2, and 4). Additionally, no significant associations between MRproADM or MRproANP and TBI were detected (Tables 2, 3 and 4).

**Table 1:** Baseline characteristics of the study population, based on sex

Sex (% of total *n* = 519)	Female (37.0)		Male (63.0)	*p* value
Age (years)	60.3 ± 3.1		60.4 ± 3.1	0.632
Diabetes duration, median, IQR (years)	6.0 (3.0–10.0)		6.0 (2.0–9.0)	0.302
BMI (kg/m²)	30.8 ± 5.4		29.2 ± 3.9	< 0.001**
Current smoking (%)	23.5		16.0	0.045*
**Medication, (%)**				
Diet only	27.6		26.9	0.864
OAD	40.6		45.9	0.245
Insulin	12.0		13.8	0.561
OAD + Insulin	19.8		13.5	0.056
Diuretics	19.3		14.3	0.013*
Beta-blockers	30.7		24.2	0.102
ACE-Is/ARBs	40.1		40.5	0.931
Calcium channel blockers	11.5		13.2	0.557
Statins	54.5		52.0	0.399
**Laboratory measures**				
eGFR (ml/min/1.7 m²)	70.7 ± 16.5		77.3 ± 16.5	< 0.001**
Cholesterol (mmol/L)	5.0 ± 1.0		4.7 ± 0.9	< 0.001**
HbA1c-IFCC (mmol/mol)	52.3 ± 11.7		52.9 ± 11.4	0.580
Copeptin, median, IQR (pmol/L)	5.9 (3.6–7.8)		8.2 (5.6–11.8)	< 0.001**
MRproADM (nmol/L)	0.6 ± 0.1		0.5 ± 0.1	< 0.001**
MRproANP (pmol/L)	67.6 ± 29.3		67.4 ± 28.8	0.974
**Physiological examination**				
Mean 24-hour ambulatory SBP (mmHg)	129.6 ± 14.4		130.7 ± 13.4	0.406
Toe brachial index	0.8 ± 0.1		0.8 ± 0.1	0.247
Aortic pulse wave velocity (m/s)	10.1 ± 2.1		10.4 ± 2.0	0.129

BMI, body mass index; OAD, oral anti-diabetic drugs; ACE-I/ARB, angiotensin-converting enzyme inhibitor; ARB, angiotensin receptors blocker; eGFR, estimated glomerular filtration rate, HbA1c-IFCC, glycated hemoglobin; SBP, systolic blood pressure

Missing value: 29 diabetes duration, 10 smoking status, 2 diuretics, 1 ACE-Is/ARBs, 2 calcium channel blockers, 2 statins, 19 eGFR, 15 cholesterol, 8 HbA1c-IFCC, 25 mean 24-hour ambulatory SBP

**Association is significant at the 0.001 level (2-tailed)

*Association is significant at the 0.05 level (2-tailed)

### Aortic pulse wave velocity

No significant associations were found between sex, smoking status, cholesterol, or MRproANP level and aPWV (Table 2). After adjustment, only age, BMI, eGFR, mean 24-hour SBP, HbA1c, and copeptin remained significantly associated with aPWV (Tables 3 and 4).

### Biomarkers and arterial disorders

Table 2 shows that copeptin and MRproADM were associated with aPWV, while only copeptin was associated with TBI. MRproANP was not associated with TBI or aPWV (Tables 2, 3 and 4). After adjusting for sex, age, diabetes duration, BMI, smoking status, HbA1c, total cholesterol, eGFR, and mean 24-hour SBP, only copeptin remained significantly associated with TBI and aPWV. These results remained unchanged even after considering the effects of antihypertensive treatment ARBs/ACE-Is and statins (Supplementary file: Tables 1 and 2).

**Table 2:** Univariable linear regression analysis: The associations of cardiovascular risk factors and each biomarker, with the toe brachial index and aortic pulse wave velocity

	Toe brachial index			Aortic pulse wave velocity (m/s)		
	β	95.0% CI	*p* value	β	95.0% CI	*p* value
Female sex	−0.015	−0.040–0.010	0.247	10.283	−0.649–0.083	0.129
Age (years)	−0.006	−0.010– (−0.002)	0.002*	0.150	0.094–0.205	< 0.001**
Diabetes duration (years)	−0.003	−0.005– (−0.001)	< 0.001**	0.054	0.026–0.082	< 0.001**
BMI (kg/m²)	0.003	0.0002–0.006	0.037*	0.071	0.032–0.109	< 0.001**
Current smoking	−0.036	−0.067– (−0.004)	0.026*	−0.443	−0.902–0.017	0.059
eGFR (ml/min/1.7 m²)	0.001	0.0001 −0.002	0.035*	0.014	0.003–0.024	0.012*
Mean 24-hour ambulatory SBP (mmHg)	−0.001	-0.002–0.0003	0.010*	0.049	0.036–0.061	< 0.001**
Cholesterol (mmol/L)	−0.005	−0.018 − 0.007	0.394	−0.131	−0.313–0.051	0.158
HbA1c-IFCC (mmol/mol)	−0.001	−0.002– (−0.0003)	0.014*	0.026	0.010–0.041	0.001**
Copeptin (pmol/L)	−0.002	−0.003– (−0.0003)	0.017*	0.040	0.018–0.061	< 0.001**
MRproADM (nmol/L)	−0.057	−0.148–0.034	0.220	1.950	0.634–3.267	0.004*
MRproANP (pmol/L)	−0.0003	−0.001–0.0001	0.129	0.005	−0.001–0.011	0.100

**Association is significant at the 0.001 level (2-tailed)

*Association is significant at the 0.05 level (2-tailed)

**Table 3:** Multivariable linear regression analysis: The associations of each biomarker, adjusted for cardiovascular risk factors, with the toe brachial index and aortic pulse wave velocity

	Toe brachial index			Aortic pulse wave velocity (m/s)		
	β	95.0% CI	*p* value	β	95.0% CI	*p* value
Copeptin (pmol/L)	−0.002	−0.004 − (−0.001)	0.007*	0.023	0.002–0.043	0.034*
MRproADM (nmol/L)	−0.054	−0.166–0.057	0.337	0.073	−1.467–1.614	0.926
MRproANP (pmol/L)	−0.0003	−0.0008–0.0001	0.139	0.002	−0.004–0.008	0.456

Each marker was adjusted for sex, age, diabetes duration, BMI, smoking status, HbA1c-IFF, total cholesterol, eGFR, and mean 24-hour ambulatory SBP

**Association is significant at the 0.001 level (2-tailed)

*Association is significant at the 0.05 level (2-tailed)

**Table 4:** Multivariable linear regression analysis: The associations of cardiovascular risk factors and all biomarkers, with the toe brachial index and aortic pulse wave velocity

	Toe brachial index			Aortic pulse wave velocity (m/s)		
	β	95.0% CI	*p* value	β	95.0% CI	*p* value
Female sex	−0.0218	−0.0506–0.0070	0.137	0.013	−0.387–0.413	0.950
Age (years)	−0.0026	−0.0070–0.0017	0.232	0.163	0.103–0.223	< 0.001**
Diabetes duration (years)	−0.0029	−0.0049– (−0.0008)	0.006*	0.024	−0.004–0.052	0.097
BMI (kg/m²)	0.0027	−0.0005–0.0060	0.102	0.055	0.009–0.100	0.018*
Current smoking	−0.0171	−0.0516–0.0174	0.331	−0.073	−0.552–0.406	0.763
eGFR (ml/min/1.7 m²)	0.0004	−0.0004–0.0011	0.378	0.015	0.004–0.025	0.008*
Mean 24-hour ambulatory SBP (mmHg)	−0.0005	−0.0014–0.0004	0.299	0.044	0.031–0.057	< 0.001**
Cholesterol (mmol/L)	−0.0024	−0.0159–0.0112	0.730	−0.139	−0.811–1.138	0.145
HbA1c-IFCC (mmol/mol)	−0.0007	−0.0018–0.0005	0.270	0.019	0.003–0.036	0.020*
Copeptin (pmol/L)	−0.0020	−0.0035– (−0.0005)	0.010*	0.023	0.002–0.044	0.035*
MRproADM (nmol/L)	−0.0094	−0.1287–0.1099	0.878	−0.366	−2.021–1.289	0.664
MRproANP (pmol/L)	−0.0003	−0.0008–0.0002	0.232	0.002	−0.004–0.009	0.460

**Association is significant at the 0.001 level (2-tailed)

*Association is significant at the 0.05 level (2-tailed)

## Discussion

In this study, we found a significant association between copeptin, aPWV, and TBI in middle-aged patients with type 2 DM, independent of sex, age, diabetes duration, BMI, smoking status, HbA1c, total cholesterol, eGFR, and mean 24-hour SBP. However, no such association was found with MRproADM and MRproANP. This result is novel; to our knowledge, no other study has yet examined the associations between aPWV, TBI, mean 24-hour ambulatory SBP, MRproADM, MRproANP, and copeptin simultaneously. Our research focused on two arterial disorder indicators, the aPWV and TBI, which denote different structural characteristics of the vascular wall in various branches of the arterial tree. Moreover, we explored their relationships with three biomarkers: copeptin, MRproADM, MRproANP, and 24-hour ambulatory SBP.

It is worth noting that aPWV and TBI are two different indices that do not exhibit significant Spearman correlations and only demonstrate a modest inverse correlation according to Pearson’s correlation, *r* = − 0.108 (Supplementary file: Table 3a and 3b). However, we found that copeptin is associated with aPWV and TBI, which are known to have predictive value for CVD [22, 23]. This suggests that they may share similar underlying causes, such as age, sex, smoking status, hypertension, hypercholesterolemia, and diabetes. The link between copeptin and CVD in patients with type 2 DM is thought to be due to chronic hyperglycemia causing damage to blood vessels, leading to arterial stiffness [24–26]. This highlights the potential importance of measuring copeptin levels in patients with a greater risk of CVD to help identify potential health risks.

Arterial stiffness is a common risk factor for CVD among individuals with diabetes [22, 27]. The aPWV is considered the most reliable standard for measuring arterial stiffness [16] and was a strong predictor of CV events in the CARDIPP population [22]. At the same time, copeptin has also been shown to predict the incidence of CVD and total mortality in patients with type 2 diabetes treated in primary care [28–30]. However, these copeptin-related studies did not consider vascular disorder measurements when examining conventional CVD risk markers. Our research indicated that copeptin is linked to aPWV, a marker of central aortic stiffness, as shown by Schill et al. [31], and to TBI, a marker of PAD.

It has been suggested that MRproADM, MRproANP, and copeptin are potentially linked to microvascular endothelial dysfunction [15, 17, 32]. While copeptin has been related to the long-term development of symptomatic PAD, no such association has been noted for MRproANP or MRproADM [33]. MRproANP has also been proposed as a marker of arterial stiffness and severity of hypertension [34]. Our study found a correlation between MRproANP and the mean 24-hour ambulatory SBP (*r* = 0.094, *p* = 0.038), but no association was found between MRproANP and aPWV (Supplementary file: Table 3a and 3b). On the other hand, the mean of 24-hour ambulatory SBP was associated with copeptin, TBI, and aPWV. After adjusting for other risk factors, the mean of 24-hour ambulatory SBP remained significantly associated with aPWV (β = 0.044, *p* < 0.001) but not with TBI (Tables 2 and 4). This indicates that blood pressure is important for aPWV but not for TBI.

It has also been found that distal arteries do not stiffen with age [35]. Besides vascular smooth muscle cells (VSMCs), large proximal arteries contain many elastic lamellae. In contrast, VSMCs are more common in the media of distal arteries [36]. Vasopressin, which acts through V1aR, can cause platelet aggregation and vasoconstriction in VSMCs [15]. High vasopressin levels, as indicated by copeptin, may lead to hypertension and affect vascular smooth muscle, which can cause premature vascular aging [16]. As a result, distal arteries may stiffen regardless of age [35]. Our findings support this idea, as TBI is not affected by age, but aPWV is.

Adjusting for antihypertensive medication, such as angiotensin-converting enzyme inhibitors or angiotensin II receptor I antagonists and statins did not change the results, as shown a previous study [37].

### Strengths and limitations

This study has several strengths. First, the study included a relatively large cohort recruited from several health centers with broad inclusion criteria, making it representative of patients typically seen in primary care. Second, extensive baseline testing allowed adjustment for several potential confounders, including aPWV and TBI.

It is important to note that this study has several limitations. There was an unintentional gender imbalance, with more male participants than female, and ethnic data were not collected. The study’s cross-sectional design is a significant limitation, making it impossible to draw conclusions about causality. The analysis focused on assessing the associations between blood markers, not their predictive values, to TBI and aPWV. Nonetheless, the novelty of the study’s results is worth noting.

## Conclusion

Copeptin, a biomarker of various stresses, is associated with TBI, and aPWV, a marker of arterial disorders, independently of sex, age, diabetes duration, BMI, smoking status, HbA1c, total cholesterol, eGFR, and mean 24-hour ambulatory SBP. Measuring copeptin levels may make identifying individuals at risk for arterial disorders simpler than current methods, such as aPWV or TBI. However, further studies must be conducted to evaluate the predictive value of copeptin levels for identifying patients at increased risk of arterial disorders in primary care.

### Electronic supplementary material

Below is the link to the electronic supplementary material.

Supplementary Material

## Data Availability

The datasets used and/or analyzed in the study are available from the corresponding author upon reasonable request.

## Abbreviations

ACE-I: Angiotensin−Converting Enzyme Inhibitor
aPWV: aortic Pulse Wave Velocity
ARB: Angiotensin Receptor Blocker
BMI: Body Mass Index
SBP: Systolic Blood Pressure
CARDIPP: Cardiovascular Risk Factors in Patients with Diabetes−a Prospective Study in Primary Care
CV: Cardiovascular
CVD: Cardiovascular Disease
GFR: Glomerular Filtration Rate
HbA1c: Glycated hemoglobin
MRproADM: Midregional portion of proadrenomedullin
MRproANP: Midregional fragment of the N-terminal of proatrial natriuretic peptide
OAD: Oral Anti-diabetic Drug
PAD: Peripheral Arterial Disease
TBI: Toe Brachial Index
VSMCs: Vascular Smooth Muscle cells
