# Supplementary material for: Plasma copeptin and markers of arterial disorder in patients with type 2 diabetes, a cross-sectional study
Source: Cardiovasc Diabetol. 2024 Jun 12;23:200. doi: 10.1186/s12933-024-02291-2 (PMC11170787; doi:10.1186/s12933-024-02291-2)
Supplement: Supplementary file 1 — Supplementary Material [file 12933_2024_2291_MOESM1_ESM.docx]

**Supplementary Information:**

**Plasma copeptin and markers of arterial disorder in patients with type 2 diabetes, a cross-sectional study.**

Lee Ti Davidson^*^, Jan Engvall, Simona I. Chisalita, Carl Johan Östgren, Fredrik H Nyström

Table 1. Multivariable linear regression analysis: Association of all biomarkers, conventional CVD risk factors, ACE-Is/ARBs, and statins with TBI and aPWV.

|  | Toe brachial index | | | Aortic pulse wave velocity (m/s) | | |
| --- | --- | --- | --- | --- | --- | --- |
|  | β | 95.0% CI | *p* value | β | 95.0% CI | *p* value |
| Female sex | - 0.023 | - 0.052 – 0.006 | 0.118 | - 0.003 | - 0.406 - 0.399 | 0.988 |
| Age (years) | - 0.003 | - 0.007 – 0.002 | 0.220 | 0.163 | 0.103 – 0.223 | < 0.001** |
| Diabetes duration (years) | - 0.003 | - 0.005 – (- 0.001) | 0.006* | 0.023 | - 0.006 – 0.051 | 0.117 |
| BMI (kg/m²) | 0.003 | - 0.001 – 0.006 | 0.099 | 0.055 | 0.010 – 0.100 | 0.017* |
| Current smoking | - 0.017 | - 0.052 – 0.018 | 0.342 | - 0.094 | - 0.577 – 0.388 | 0.700 |
| eGFR (ml/min/1.7 m²) | 0.0004 | - 0.0004 – 0.0011 | 0.383 | 0.016 | 0.006 – 0.027 | 0.004* |
| Mean 24-hour ambulatory SBP (mmHg) | - 0.001 | - 0.0014 – 0.0004 | 0.303 | 0.043 | 0.030 – 0.056 | < 0.001** |
| Cholesterol (mmol/L) | - 0.001 | - 0.015 – 0.013 | 0.910 | - 0.105 | - 0.302 – 0.092 | 0.294 |
| HbA1c-IFCC (mmol/mol) | - 0.001 | - 0.0028 – 0.0005 | 0.242 | 0.018 | 0.002 – 0.034 | 0.030* |
| Copeptin (pmol/L) | - 0.002 | - 0.0035 – (- 0.0005) | 0.011* | 0.023 | 0.002 -0.044 | 0.034* |
| MRproADM (nmol/L) | - 0,009 | - 0.129 – 0.111 | 0.882 | -0.388 | -2.042 – 1.266 | 0.645 |
| MRproANP (pmol/L) | -0.0003 | - 0.0008 – 0.0002 | 0.229 | 0.002 | -0.004 – 0.009 | 0.455 |
| ACE-Is/ARBs | 0.008 | - 0.018 – 0.034 | 0.552 | 0.245 | - 0.119 – 0.609 | 0.187 |
| Statins | 0.007 | - 0.021 – 0.035 | 0.617 | 0.104 | - 0.277 – 0.486 | 0.592 |

** Association is significant at the 0.001 level (2-tailed).

* Association is significant at the 0.05 level (2-tailed).

Table 2. Multivariable linear regression analysis: Association of copeptin, conventional CVD risk factors, ACE-Is/ARBs, and statins with TBI and aPWV.

|  | Toe brachial index | | | Aortic pulse wave velocity (m/s) | | |
| --- | --- | --- | --- | --- | --- | --- |
|  | β | 95.0% CI | *p* value | β | 95.0% CI | *p* value |
| Female sex | - 0.024 | - 0.053 – 0.005 | 0.106 | - 0.018 | - 0.414 - 0.379 | 0.930 |
| Age (years) | - 0.003 | - 0.007 – 0.001 | 0.147 | 0.163 | 0.104 – 0.222 | < 0.001** |
| Diabetes duration (years) | - 0.003 | - 0.005 – (-0.001) | 0.006* | 0.023 | - 0.005 – 0.051 | 0.110 |
| BMI (kg/m²) | 0.003 | - 0.0005 – 0.0060 | 0.081 | 0.051 | 0.010 – 0.092 | 0.016* |
| Current smoking | - 0.017 | - 0.052 – 0.017 | 0.329 | - 0.110 | - 0.587 – 0.367 | 0.650 |
| eGFR (ml/min/1.7 m²) | 0.0004 | - 0.0004 – 0.0011 | 0.313 | 0.017 | 0.006 – 0.027 | 0.003* |
| Mean 24-hour ambulatory SBP (mmHg) | - 0.001 | - 0.002 – 0.0004 | 0.242 | 0.043 | 0.030 – 0.056 | < 0.001** |
| Cholesterol (mmol/L) | - 0.001 | - 0.016 – 0.013 | 0.861 | - 0.100 | - 0.297 – 0.096 | 0.316 |
| HbA1c-IFCC (mmol/mol) | - 0.0007 | - 0.0018 – 0.0005 | 0.270 | 0.018 | 0.002 – 0.034 | 0.030* |
| Copeptin (pmol/L) | - 0.002 | - 0.004 – (- 0.001) | 0.008* | 0.023 | 0.002 -0.043 | 0.034* |
| ACE-Is/ARBs | 0.007 | - 0.019 – 0.034 | 0.582 | 0.248 | - 0.115 – 0.611 | 0.180 |
| Statin | 0.007 | - 0.021 – 0.035 | 0.616 | 0.108 | - 0.273 – 0.488 | 0.579 |

** Association is significant at the 0.001 level (2-tailed).

* Association is significant at the 0.05 level (2-tailed).

Table 3a-b. Correlation analysis between copeptin, MRproADM, MRproANP, mean 24-hour ambulatory SBP, TBI, and aPWV.

1. Pearson correlation

|  | MRPROADM nmol/l | MRPROANP pmol/l | TBI | aPWV m/s | Mean 24-hour ambulatory SBP mmHg |
| --- | --- | --- | --- | --- | --- |
| COPAVP pmol/L | *r*= 0.158**  *p* <.001  CI 0.073–0.241 | *r* = 0.078  *p* = 0.075  CI -0.008–0.0163 | *r* = -0.105*  *p* = 0.017  CI -0.189– (-0.019) | *r* = 0.159**  *p* <.001  CI 0.074–0.242 | *r* = 0.128**  *p* = 0.004  CI -0.203– (-0.028) |
| MRPROADM nmol/L | | *r* = 0.368**  *p* <.001  CI 0.291–0.440 | *r* = -0.054  *p* = 0.22  CI -0.139–0.032 | *r* = 0.127**  *p* = 0.004  CI 0.041–0.211 | *r* = 0.156**  *p* <.001  CI 0.069–0.241 |
| MRPROANP pmol/L | | | *r* = -0.067  *p* = 0.129  CI -0.152–0.020 | *r* = 0.072  *p* = 0.100  CI -0.014–0.157 | *r* = 0.094*  *p* <.038  CI 0.005–0.180 |
| TBI | | | | *r* = - 0.108*  *p* = 0.013  CI -0.193– (-0.023) | *r* = -0.116**  *p* <.010  CI -0.203– (-0.028) |
| aPWV m/s | | | | | *r* = 0.322**  *p* <.001  CI 0.241–0.399 |
| ** Correlation is significant at the 0.01 level (2-tailed). | | | | | |
| * Correlation is significant at the 0.05 level (2-tailed). | | | | | |

1. Spearman correlation coefficients

|  | MRPROADM nmol/l | MRPROANP pmol/l | TBI | aPWV m/s | Mean 24-hour ambulatory SBP mmHg |
| --- | --- | --- | --- | --- | --- |
| COPAVP pmol/L | *r_s_*  = 0.280**  *p* <.001  CI 0.197–0.360 | *r_s_* = 0.090*  *p* = 0.040  CI 0.001–0.177 | *r_s_* = -0.021  *p* = 0.627  CI -0.110–0.067 | *r_s_* = 0.129*  *p* = 0.003  CI 0.041–0.215 | *r_s_* = 0.169**  *p* = <.001  CI 0.079–0.256 |
| MRPROADM nmol/L | | *r_s_* = 0.340**  *p* <.001  CI 0.259–0.416 | *r_s_* = -0.040  *p* = 0.368  CI -0.128–0.049 | *r_s_* = 0.154**  *p* = <.001  CI 0.066–0.239 | *r_s_* = 0.156**  *p* <.001  CI 0.06–0.244 |
| MRPROANP pmol/L | | | *r_s_* = -0.021  *p* = 0.626  CI -0.110–0.067 | *r_s_* = 0.079  *p* = 0.072  CI -0.010–0.166 | *r_s_* = 0.079  *p* = 0.079  CI -0.012–0.169 |
| TBI | | | | *r_s_* = -0.076  *p* = 0.085  CI -0.163–0.013) | *r_s_* = -0.105*  *p* = 0.020  CI -0.194– (-0.014) |
| aPWV m/s | | | | | *r_s_* = 0.334**  *p* <.001  CI 0.251–0.413 |
| ** Correlation is significant at the 0.01 level (2-tailed). | | | | | |
| * Correlation is significant at the 0.05 level (2-tailed). | | | | | |
